# Stability, Digestion, and Cellular Transport of Soy Isoflavones Nanoparticles Stabilized by Polymerized Goat Milk Whey Protein

**DOI:** 10.3390/antiox13050567

**Published:** 2024-05-03

**Authors:** Mu Tian, Jianjun Cheng, Mingruo Guo

**Affiliations:** 1 College of Food Science and Technology, Southwest Minzu University, Chengdu 610041, China; 80300200@swun.edu.cn; 2 Key Laboratory of Dairy Science, Northeast Agricultural University, Harbin 150030, China; jjcheng@neau.edu.cn; 3 Department of Nutrition and Food Sciences, College of Agriculture and Life Sciences, University of Vermont, Burlington, VT 05405, USA

**Keywords:** nanoparticles, stability, gastrointestinal digestion, caco-2 cells, bioavailability

## Abstract

Soy isoflavones (SIF) are bioactive compounds with low bioavailability due to their poor water solubility. In this study, we utilized polymerized goat milk whey protein (PGWP) as a carrier to encapsulate SIF with encapsulation efficiency of 89%, particle size of 135.53 nm, and zeta potential of −35.16 mV. The PGWP-SIF nanoparticles were evaluated for their stability and in vitro digestion properties, and their ability to transport SIF was assessed using a Caco-2 cell monolayer model. The nanoparticles were resistant to aggregation when subjected to pH changes (pH 2.0 to 8.0), sodium chloride addition (0–200 mM), temperature fluctuations (4 °C, 25 °C, and 37 °C), and long-term storage (4 °C, 25 °C, and 37 °C for 30 days), which was mainly attributed to the repulsion generated by steric hindrance effects. During gastric digestion, only 5.93% of encapsulated SIF was released, highlighting the nanoparticles’ resistance to enzymatic digestion in the stomach. However, a significant increase in SIF release to 56.61% was observed during intestinal digestion, indicating the efficient transport of SIF into the small intestine for absorption. Cytotoxicity assessments via the MTT assay showed no adverse effects on Caco-2 cell lines after encapsulation. The PGWP-stabilized SIF nanoparticles improved the apparent permeability coefficient (*Papp*) of Caco-2 cells for SIF by 11.8-fold. The results indicated that using PGWP to encapsulate SIF was an effective approach for delivering SIF, while enhancing its bioavailability and transcellular transport.

## 1. Introduction

Soy isoflavones, primarily extracted from soybeans and their derivatives [1], have been investigated for several health benefits. They include improving antioxidative activity, which aids in combating diseases related to oxidative stress [2]. Soy isoflavones may reduce the risk of breast cancer by potentially inhibiting cancer cell growth and proliferation [3]. Additionally, they show promise in alleviating menopausal symptoms due to their estrogen-like effects [4]. Moreover, soy isoflavones have been associated with preventing osteoporosis by promoting bone health and reducing bone loss [5]. Because of their health benefits, SIF have been recommended as a functional ingredient for formulation of healthy foods and pharmaceutical products. In a study conducted by Liu et al. [6], it was reported that soy milk enriched with soy isoflavones demonstrated enhanced nutritional content and improved bioavailability of these bioactive compounds. Recent research also suggests that soy isoflavones have the potential to stimulate the growth of probiotics and enhance their antibacterial activity in vitro [7]. Additionally, a study observed favorable prognostic outcomes in Chinese early-stage breast cancer survivors with moderate soy isoflavone intake [8].

Despite their diverse bioactivities, soy isoflavones have poor bioavailability, primarily attributed to their water insolubility, susceptibility to chemical damages, and reduced effectiveness when administered orally [9]. The poor solubility limits their effectiveness when administered orally, owing to their poor solubility and absorption in the gastrointestinal tract. Consequently, the bioavailability of these oral bioactive substances in the human body is extremely low, hindering their utilization in various applications such as food, dietary supplements, and pharmaceuticals. These challenges emphasize the need for innovative approaches to enhance the solubility and stability of soy isoflavones to maximize their full potential in improving human health.

Nano-delivery systems have gained attention due to their ability to overcome these limitations by improving solubility, enhancing stability, and offering sustained release characteristics for bioactive compounds [10,11,12]. Additionally, bioactive substances encapsulated in nanoparticles have been found to exhibit resilience to environmental factors such as light and heat [13]. Moreover, the protective capabilities of these nanocarriers ensure the preservation of encapsulated substances during gastrointestinal digestion, thereby enhancing their bioavailability and potential health benefits. Consequently, nano-delivery systems are considered optimal candidates for enhancing the bioavailability of polyphenols. Previous efforts to improve the bioavailability of soy isoflavones have involved the development of various delivery systems for their encapsulation. These systems include mesoporous starch microcapsules [14], sodium carboxymethyl cellulose capsules [15], and soy polysaccharide hydrogel [16]. However, utilization of proteins, particularly milk proteins, as delivery carriers for encapsulating soy isoflavones has been relatively limited. Liu et al. [17] demonstrated the potential of milk protein as a promising carrier for encapsulating SIF with high encapsulation efficiency.

Goat milk whey proteins offer the advantages of high nutritional value and excellent functional properties [18]. Li et al. [19] introduced an innovative approach involving ultrasound-assisted Maillard reaction to enhance the functional properties of goat whey protein by conjugating it with gum arabic or citrus pectin. This underscores the potential for improving the functionality and applicability of goat whey protein in food systems. Additionally, Hovjecki et al. [20] discovered that fortifying goat milk yogurt with goat whey protein concentrate enhanced its texture, flavor, and stability during storage, providing valuable insights into the practical applications of goat whey protein in dairy products. Whey proteins are amphiphilic molecules with great surface activity and emulsifying properties. The construction of nano-delivery systems using whey proteins primarily relies on electrostatic attraction, hydrophobic interaction, and hydrogen bonding effects [21,22]. Another notable benefit of whey protein is its ability to serve as a stabilizer. Its resistance to pepsin digestion and susceptibility to be degraded by trypsin may make whey protein-based nanoparticles suitable to be used as controlled-release delivery systems [23].

In our previous studies, we utilized polymerized goat milk whey protein to encapsulate soy isoflavones to improve the solubility [18]. However, application of these systems in food products is often constrained by environmental factors such as pH, ionic strength, and temperature [24]. In this study, we investigated the stability of PGWP-SIF nanoparticles under varying pH, ionic strength, temperature, and storage time to assess their performance in gastrointestinal digestion. Additionally, we examined the digestion properties of the nanoparticles, including their stability during digestion and controlled release of soy isoflavones, using in vitro digestion models. Furthermore, we evaluated the cytotoxicity and bioavailability of PGWP-SIF nanoparticles using Caco-2 cells. The findings are expected to provide valuable insights for the efficient utilization of soy isoflavones in functional foods.

## 2. Materials and Methods

### 2.1. Materials

Soy isoflavones (SIF) sample with a purity of 80% was purchased from Beijing Solarbio Science and Technology Co., Ltd. (Beijing, China). Bile salts, pepsin (3000 U/mg), and pancreatin (200 U/mg) were sourced from Sigma-Aldrich (St. Louis, MO, USA). Dulbecco’s Modified Eagle Medium (DMEM) was obtained from Gibco (Shanghai, China). The Alkaline Phosphatase (ALP) Assay Kit was acquired from the Beyotime Institute of Biotechnology (Beijing, China). The Caco-2 cell line was provided by the Shanghai Cell Bank (Shanghai, China). All other chemicals used were of analytical grade.

### 2.2. Preparation of Polymerized Goat Milk Whey Protein-Loaded Soy Isoflavones (PGWP-SIF) Nanoparticles

Goat milk whey protein was prepared using membrane separation technology, as detailed in our prior work [18]. This product consists of 80.99% protein, 18.67% lactose, and 0.34% ash. We followed the procedures of our previous study [18] to prepare PGWP and SIF solutions. To obtain a 10% (*w*/*v*) goat milk whey protein solution, the whey protein powder was dissolved in deionized water and stored at 4 °C for a 12-h period to ensure complete hydration. Subsequently, the pH was adjusted to 7.7 using 1 M sodium hydroxide, followed by heating to 75 °C for 25 min with continuous stirring. The solution was then rapidly cooled to room temperature, resulting in the formation of polymerized goat milk whey protein (PGWP).

Soy isoflavones (SIF) were dissolved in 70% ethanol (5 mg/mL) with stirring using a magnetic stirrer (IKA, Staufen, Germany) and heated to 50 °C for 1 h to produce a clear solution. This solution was shielded from light by wrapping the container with aluminum foil.

The PGWP-SIF solution was prepared by combining PGWP solution, SIF solution, and deionized water to achieve a final SIF concentration of 2.4 mg/mL, while maintaining the PGWP content at 40 mg/mL. The mixture was stirred for 2 h to establish a stable system. Subsequently, the combined solution was treated to remove ethanol using nitrogen gas, and deionized water was added to maintain the original volume. The resulting solution was stored in darkness following the procedure described in our prior work [18]. We prepared goat milk whey protein-soy isoflavones nanoparticles (GWP-SIF) with an equivalent amount of SIF using the same methodology as a comparison.

### 2.3. Stability Analysis

#### 2.3.1. Effect of Different pH on the Stability

The freshly prepared nanoparticle dispersions were adjusted to the desired pH values (2.0, 3.0, 4.0, 6.0, 7.0, 8.0) using 1 M NaOH or HCl solutions. Particle size and zeta potential were carried out using a Malvern Zetasizer Nano ZS90 (Malvern Instruments Ltd., Worcestershire, UK), as described by Gelebart et al. [25]. Sample solutions were diluted to 0.1% (*w*/*v*) with demineralized water and stored at 25 °C for 30 min to ensure equilibrium. The refractive indexes for protein and water were 1.450 and 1.333, respectively. All measurements were performed in triplicate. 

#### 2.3.2. Effect of Ionic Strength on the Stability

The ionic strength stability of the nanoparticles was evaluated using a modified method based on a previous study [26]. The freshly prepared nanoparticle dispersions were mixed with equal volumes of NaCl solutions at ionic strengths of 0, 100, 200, 300, and 400 mM to create a series of samples with varying salt concentrations (0, 50, 100, 150, 200 mM). After allowing the samples to reach equilibrium for 12 h, the particle size and zeta potential of the nanoparticle dispersions were measured.

#### 2.3.3. Effect of Temperature on the Stability

The temperature stability of the nanoparticles was assessed by a previous study with some modifications [27]. The samples were incubated in a thermostatic water bath at various temperatures of 25 °C and 37 °C for 2 h, and at 4 °C in a refrigerator for the same duration. These temperature ranges were selected to mimic common environmental conditions encountered during food production, transportation, and storage. Specifically, incubating at 25 °C represents room temperature storage conditions, while 37 °C simulates physiological conditions similar to those in the human body, and 4 °C reflects typical refrigeration conditions. Following incubation, the samples were returned to room temperature, and the particle size and zeta potential were determined.

#### 2.3.4. Effect of Storage Time on the Stability

The storage stability of the nanoparticles was evaluated by observing samples over a period of 30 days at different temperatures of 4 °C, 25 °C, and 37 °C. At regular intervals, samples stored at these temperatures were withdrawn, and the particle size and zeta potential of the nanoparticles were measured.

### 2.4. Antioxidant Activity 

#### 2.4.1. DPPH Radical Scavenging Activity

The DPPH scavenging capacity of the samples was assessed following a previously described method with slight modifications [28]. Briefly, 2 mL of free SIF, GWP-SIF, PGWP-SIF nanoparticles, and ultrapure water (control group) were added to 2 mL of DPPH⋅solution (100 μM) and stored at room temperature in the dark for 30 min. The absorbance of the solution was measured at 517 nm using an ultraviolet–visible spectrophotometer (UV-2600, Shimadzu, Kyoto, Japan). DPPH radical scavenging activity was calculated using the following equation:

DPPH radical Scavenging activity (%) = (1 − A_sample_/A_control_) × 100 (1)

#### 2.4.2. ABTS Radical Scavenging Activity 

The ABTS scavenging capacity of the samples was assessed using a method previously described [29]. The ABTS solution (7 mM) was combined with potassium persulfate solution (2.5 mM) in equal volumes to prepare a working solution. This working solution was incubated at 25 °C for 16 h in the dark. The absorbance of the ABTS working solution was adjusted to a value of 0.70 ± 0.02 at 734 nm using PBS (10 mM, pH 7.4). The ABTS working solution (6.0 mL) was mixed with 2.0 mL of free SIF, GWP-SIF, PGWP-SIF nanoparticles, and ultrapure water (control group) and reacted for 6 min. The absorbance was determined at 734 nm. The ABTS radical scavenging activity was calculated using the following equation:

ABTS radical scavenging activity (%) = (1 − A_sample_/A_control_) × 100 (2)

### 2.5. In Vitro Digestion

The in vitro release profiles of SIF from the nanoparticles under simulated gastrointestinal conditions were investigated using a method based on prior research with minor modifications [28].

#### 2.5.1. Peptic Digestion

A 30 mL volume of simulated gastric fluid containing 3.2 mg/mL of pepsin was preheated to 37 °C and then mixed with the initial mixtures at a 1:1 mass ratio. The mixture was adjusted to pH 2.0 using 0.1 M of HCl and placed in an incubated shaker with stirring at 100 rpm at 37 °C for 2 h to simulate gastric digestion [28]. At intervals of 20, 40, 60, 80, 100, and 120 min, 0.1 mL of digesta was withdrawn. The collected samples were subsequently heated to deactivate pepsin by maintaining them at 90 °C for 5 min.

#### 2.5.2. Intestinal Digestion

After gastric digestion, the pH gastric simulation sample was adjusted to 7.0 with 0.1 M of NaOH. Then, 60 mL of simulated intestinal fluid containing 5.0 mg/mL of bile salt, 10 mM of CaCl_2_, 150 mM of NaCl, and 1.0 mg/mL of pancreatic enzymes was added to 30 mL of the digesta [30]. The pH of the mixture was readjusted to 7.0 using 0.1 M of NaOH. The mixture was then stirred at 100 rpm and 37 °C for 2 h to simulate small intestine digestion. The mixture was stirred at 100 rpm and maintained at 37 °C for 2 h to simulate small intestine digestion. Samples were collected at 20, 40, 60, 80, 100, and 120 min, respectively. After in vitro digestion, the samples were heated in a water bath at 90 °C for 5 min to deactivate the pancreatin.

The collected solutions were centrifuged at 10,000× *g* for 30 min, and the supernatant was collected to obtain the mixed micelle phase. Subsequently, 2 mL of the digested samples, both before and after centrifugation, were dissolved in 8 mL of dimethyl sulfoxide (DMSO) for SIF extraction. The content of SIF was determined by High-Performance Liquid Chromatography (HPLC). The release rate of SIF was calculated using the following equation [30]:

Release rate of SIF (%) = m_Release_/m_Initial_ × 100% (3)

where m_Initial_ and m_Release_ represent initial content of SIF and SIF released in digestive fluid, respectively.

#### 2.5.3. High-Performance Liquid Chromatography (HPLC) Analysis

**Figure 1:**
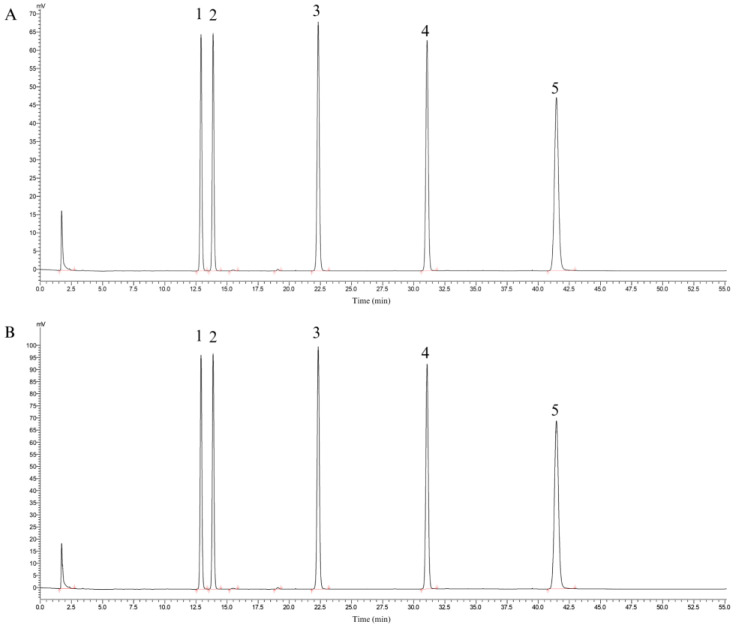
The content of each component of soy isoflavones. ((**A**): Liquid phase diagram of standard, (**B**): Liquid phase diagram of each component of soy isoflavones. 1. Daidzin; 2. Glycitin; 3. Genistin; 4. Daidzein; 5. Genistein).

Soy isoflavones were identified following the methods developed by Ogita et al. [31] with some modifications. SIF analysis was performed using an HPLC system (HPLC, UltiMate 3000, ThermoFisher Scientific, Waltham, MA, USA) equipped with a PDA UV–vis absorption detector and a C18 analytical column (150 × 4.6 mm i.d., 5 µm, YMC, Co., Ltd., Kyoto, Japan). The flow rate was maintained at 1.0 mL/min at room temperature. The injection volume was 10 µL, and the detection wavelength was set at 260 nm. The chromatography conditions were as follows: solvent A, acetonitrile; solvent B, phosphoric acid aqueous solution (pH = 3.0). SIF standards were prepared by dissolving concentrated SIF in 50% DMSO. Samples were passed through a 0.22 μm pre-evaluation filter. A serial dilution was performed to generate a standard curve, and the coefficient of determination (R_2_) was subsequently obtained. The soy isoflavones exhibited a purity of 80.45 ± 0.36% and were primarily composed of daidzin, genistin, genistein, daidzein, and glycitin. The standard curves of the five components are shown in Table 1. The ratio of the five components of soy isoflavones was 4.70:2.80:0.90:0.90:0.70 (Figure 1).

#### 2.5.4. Transmission Electron Microscopy (TEM) 

The microstructure of the samples was obtained using transmission electron microscopy (H-7650, Hitachi High-Technologies, Tokyo, Japan) as described by Ghorbani Gorji et al. [32]. The nanoparticles sample, and after simulated gastric fluid and simulated intestinal fluid digestion were diluted to appropriate concentration, was placed on carbon copper and dyed with a negative staining method. The sample was air-dried before imaging.

### 2.6. Cytotoxicity

#### 2.6.1. Cell Culture

The investigation into nanoparticle cellular transport was executed using human colon carcinoma cells (Caco-2). Caco-2 cells were cultured in Dulbecco’s Modified Eagle Medium (DMEM) with high glucose and L-glutamine, supplemented with 1% (*v*/*v*) penicillin/streptomycin and 10% (*v*/*v*) fetal bovine serum (FBS) [33]. The Caco-2 cells were maintained in an incubator under the conditions of 95% air, 5% CO_2_, and 95% humidity. The growth medium was changed every two days, and subculturing was initiated when cellular confluence reached 90%.

#### 2.6.2. MTT Cytotoxicity Assay

The cytotoxicity of the nanoparticles was assessed using methyl thiazolyl tetrazolium (MTT) assay based on a previous study with slight modifications [34]. Caco-2 cells were seeded into 96-well plates at a density of 2.5 × 10^4^ cells and incubated at 37 °C in a 5% CO_2_ environment for 72 h. After this incubation, the culture medium was removed, and 200 μL of medium containing varying concentrations of nanoparticles was added to the wells, with the medium serving as a control. After 24 h of incubation, wells were washed three times with phosphate-buffered saline (PBS). Subsequently, 200 µL of MTT-containing medium (containing 5 mg/mL of MTT in DMEM) was added to each well, and the plates were incubated at 37 °C for 4 h. The culture medium was then removed, and 150 µL of dimethyl sulfoxide (DMSO) was added to dissolve the formed formazan crystals. The absorbance was measured at 570 nm using a microplate reader (Synergy HT, BioTek, Winooski, VT, USA). The viability of cells was estimated by comparing the absorbance values of the nanoparticle-treated cells to those of the control cells [34].

#### 2.6.3. Caco-2 Cell Monolayer Incubation

Caco-2 cells were seeded onto polyethylene terephthalate (PET) filters within 6-well transwell plates (0.4 μm pore size, Corning, Somerville, MA, USA) at a density of 2.0 × 10^5^ cells per well. Subsequently, 0.5 mL of culture medium was added to the apical chamber, while 1.5 mL of medium was seeded onto the basolateral chamber [35]. The incubation medium was changed on the 5th day following seeding and subsequently every 2 days, continuing for a total of 21 days to cultivate a fully differentiated intestinal cell monolayer. Transepithelial electrical resistance (TEER) measurements were conducted using a Millicell ERS-2 Voltohmmeter (Merck, Burlington, VT, USA) to monitor the integrity of the monolayer. Additionally, the activity of alkaline phosphatase (ALP) was assessed on the 7th, 14th, and 21st days as an additional measure to confirm monolayer integrity. 

### 2.7. Cellular Transport Study

Cell transport was conducted with modifications based on previous investigations [36]. The differentiated Caco-2 monolayers were washed with Hanks buffer three times. Both SIF nanoparticles and free SIF (in DMSO) were diluted with DMEM to a final concentration of 24 µg/mL and added to the chambers. The transportation studies were carried out at 37 °C. A volume of 0.5 mL of the sample was added to the apical side, while 1.5 mL of DMEM was added to the basal side. At intervals of 0, 0.5, 1, 2, 3, and 4 h, a 0.5 mL sample was withdrawn from the basolateral side, and the withdrawn volume was replaced with HBSS (pH 7.4) each time. The apparent permeability coefficient (*Papp*, cm/s) was calculated as follows [29]:

(4) $$\mathrm{Papp}=\frac{\mathrm{dQ}}{\mathrm{dt}}\times \frac{1}{AC_{0}}$$

where dQ/dt is the mass transport rate (μg/s), A is the surface area of the transwell insert (1.12 cm^2^), and C_0_ is the initial SIF concentration in the chamber (μg/mL).

### 2.8. Statistical Analysis

Data of experiments in triplicates were statistically analyzed and presented as mean ± standard deviation. Analysis of variance (*p* < 0.05) and Duncan’s test were performed by SPSS software (version 21.0, SPSS Inc., Chicago, IL, USA).

## 3. Results and Discussion

### 3.1. Stability under Various Environmental Conditions

Nanoparticle delivery systems may encounter various environmental conditions when applied in commercial products [26]. This study assessed the impact of multiple environmental factors, including pH, ionic strength, and temperature on the stability of the nanoparticles.

#### 3.1.1. Effect of pH 

**Figure 2:**
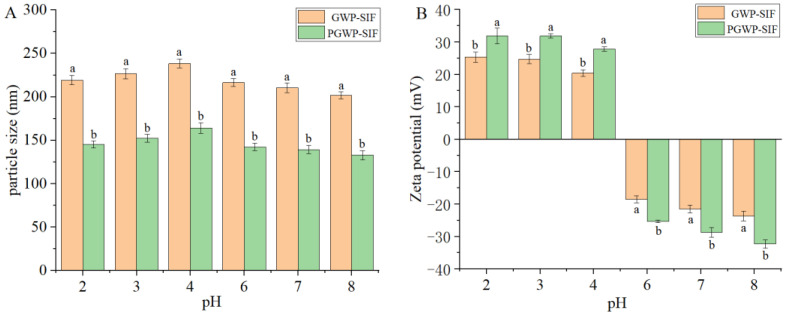
Effects of pH values (2.0–8.0) on particle size (**A**) and zeta potential (**B**) of GWP−SIF and PGWP−SIF nanoparticles. Different letters in each group are recognized as significant differences (*p* < 0.05).

Nanoparticle delivery systems may experience various environmental conditions in commercial products, including drastic pH changes within the human gastrointestinal tract after oral ingestion. Therefore, it is imperative for nano-delivery systems to exhibit strong stability to withstand pH fluctuations during food production and gastrointestinal digestion. The stability of the GWP-SIF and PGWP-SIF nanoparticles was investigated by monitoring changes in particle size and zeta potential under varying pH conditions (Figure 2). Both nanoparticles, characterized by their relatively small particle sizes, exhibited remarkable stability against aggregation within the pH range of 2 to 8, except at pH 5, where particle precipitation was observed (Figure 2A). The zeta potential exhibited a transition from highly positive values at low pH levels to highly negative values at high pH levels (Figure 2B). As the pH increased from 2 to 4, a gradual increase in particle size was observed. Notably, the isoelectric point of whey protein is approximately at pH 4.6, indicating a diminished electrostatic repulsion between the nanoparticles around this pH value, thereby accounting for the observed increase in particle aggregation. Conversely, with increasing pH from 6 to 8, the particle size exhibited a gradual decrease. The reduction at higher pH values can be attributed to the strong electrostatic repulsion between the nanoparticles, owing to their highly charged state [37]. Our findings reveal that under diverse pH conditions, PGWP-SIF nanoparticles maintained excellent water solubility and could be uniformly dispersed in aqueous environments without aggregation or precipitation. This stability of PGWP-SIF nanoparticles is crucial as it helps protect SIF from degradation, particularly in acidic environments. Furthermore, the strong acidic stability of PGWP-SIF nanoparticles suggests improved gastric digestive stability, enhancing their suitability for oral delivery.

#### 3.1.2. Effect of Ionic Strength 

**Figure 3:**
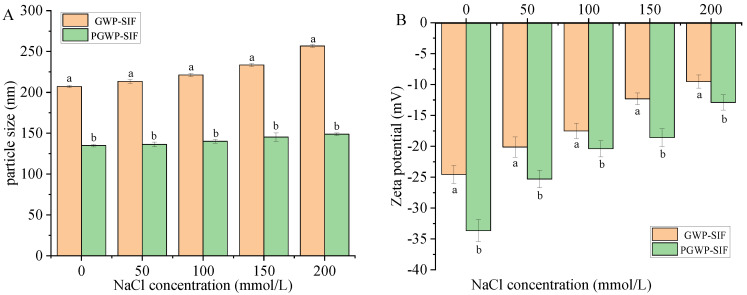
Effect of ion strength (0–200 mmol/L) on the particle size (**A**) and zeta potential (**B**) of GWP−SIF and PGWP−SIF nanoparticles. Different letters in each group are recognized as significant differences (*p* < 0.05).

The impact of sodium chloride concentration on the particle size and zeta potential of both nanoparticles was investigated and is presented in Figure 3. As the ionic strength increased, a slight increase in the particle size of both nanoparticles was observed (Figure 3A), accompanied by a decrease in the absolute zeta potential (Figure 3B). This phenomenon can be attributed to the increase in ions within the nanoparticle suspension, resulting in electrostatic shielding [26]. The charged nanoparticles underwent neutralization by counter ions, ultimately leading to a reduction in the absolute zeta potential value [38]. In addition, under conditions of elevated ionic concentration, the negative charge presented on the nanoparticle surface diminished, weakening the electrostatic repulsion forces, thereby promoting nanoparticle aggregation and an increase in particle size. Importantly, both nanoparticles exhibited favorable water solubility across varying ionic strength conditions and remained stably dispersed within aqueous environments, without experiencing nanoparticle precipitation. 

#### 3.1.3. Effect of Temperature

**Figure 4:**
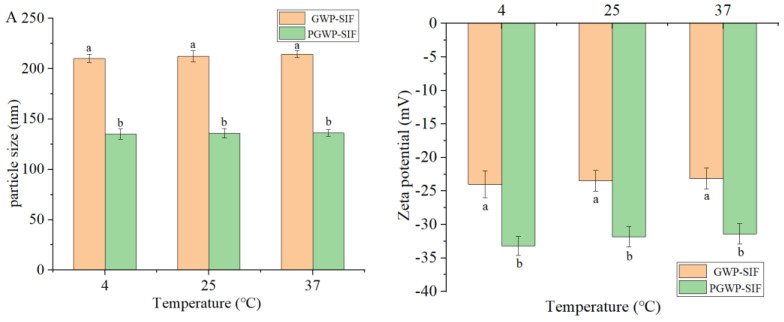
Effects of temperature on particle size (**A**) and zeta potential (**B**) of GWP−SIF and PGWP−SIF nanoparticles. Different letters in each group are recognized as significant differences (*p* < 0.05).

In the processing, transportation, and storage of food products, it is crucial that functional food constituents exhibit stability across a wide range of temperature profiles [39]. The temperature stability of both nanoparticles is shown in Figure 4. As the treatment temperature increased, a slight increase in the particle size was observed (Figure 4A), accompanied by a gradual reduction in the negative surface charge (Figure 4B). The phenomena can be attributed to the influence of temperature treatments on Brownian motion [40]. A study conducted by Liu et al. [41] also showed similar results, as polysaccharides-stabilized resveratrol nanoparticles maintained a stable structure at different temperatures of 4 °C, 25 °C, and 37 °C. This stability might be attributed to the interaction between polyphenols and biomacromolecules. In our study, the hydrophobic interaction between SIF and protein may have contributed to the temperature stability of the nanoparticles [18], enabling them to maintain stable nanostructures under different temperature conditions. The results indicated that both nanoparticles exhibited excellent temperature stability. The observed temperature stability of protein-based nanoparticles holds significant practical implications for their utilization in various applications, particularly in food technology and pharmaceuticals.

#### 3.1.4. Effect of Storage Time

The study investigated the stability of nanoparticles stored at temperatures commonly encountered during formulation, processing, and storage, namely 4 °C, 25 °C, and 37 °C, respectively, over a 30-day period (Table 2). The results demonstrated that the nanoparticles remained relatively stable against aggregation and precipitation at all tested temperatures during storage, suggesting their suitability for use in nanoparticle-based products. Notably, storing the nanoparticles at these temperatures extended their storage period beyond 30 days, enhancing their practical utility in real-world scenarios. A study conducted by Zhong et al. [42] investigated the stability of curcumin-loaded whey protein/hyaluronic acid nanoparticles at 4 °C for 28 days, which aligns with our findings. This storage stability enhances the potential for utilizing nanoparticle-based delivery systems to encapsulate and deliver hydrophobic active molecules, thereby advancing the development of functional food products and pharmaceutical formulations.

### 3.2. Antioxidant Capacity of the Nanoparticles

**Figure 5:**
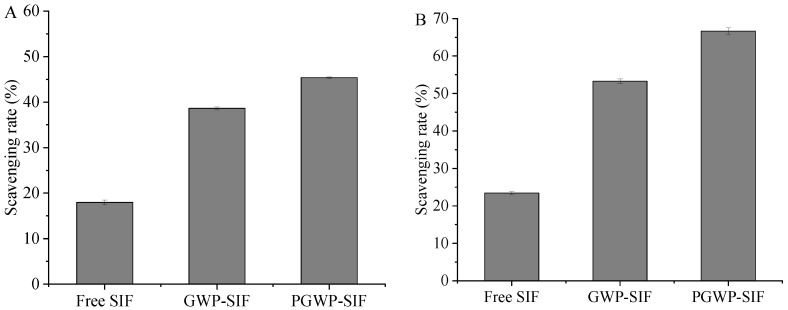
DPPH radical scavenging rate (**A**) and ABTS radical scavenging rate (**B**) of free SIF and encapsulated SIF.

The antioxidant properties of free SIF and encapsulated SIF were assessed using DPPH and ABTS scavenging assays. The DPPH radical scavenging abilities of free SIF, GWP-SIF, and PGWP-SIF nanoparticles were 18%, 39%, and 45%, respectively (Figure 5A), while the ABTS radical scavenging abilities were 23%, 53%, and 67%, respectively (Figure 5B). The enhanced antioxidant activity of PGWP-stabilized SIF nanoparticles can be attributed to the smaller particle size and larger specific surface area, which enhanced the accessibility of SIF to radicals [43]. Notably, all encapsulated SIF formulations exhibited higher scavenging rates than free SIF, indicating that the antioxidant activity of the SIF was improved after encapsulation. This observation aligned with findings by Xiao et al. [44], who concluded that nanoencapsulation is an effective technique for enhancing the antioxidant activity of bioactive compounds, such as genistein. Liu et al. [17] explored the development of whey protein nanoparticles as carriers for delivering soy isoflavones, and it was found that the antioxidant activity of soy isoflavones was significantly enhanced by encapsulation, which was consistent with our findings. Additionally, a similar effect was observed in a previous study where composite nanoparticles formed by zein, caseinate, and sodium alginate loaded with curcumin exhibited increased DPPH radical scavenging activity of curcumin [45].

### 3.3. Effects of Gastrointestinal Digestion on Nanoparticle Properties

**Figure 6:**
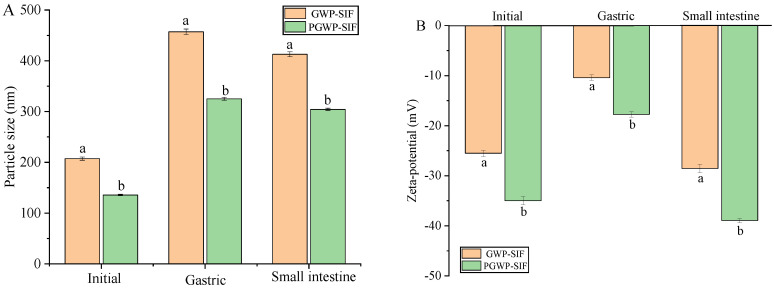
Particle size (**A**) and zeta potential (**B**) of GWP−SIF and PGWP−SIF nanoparticles during in vitro digestion. Different letters in each group are recognized as significant differences (*p* < 0.05).

**Figure 7:**
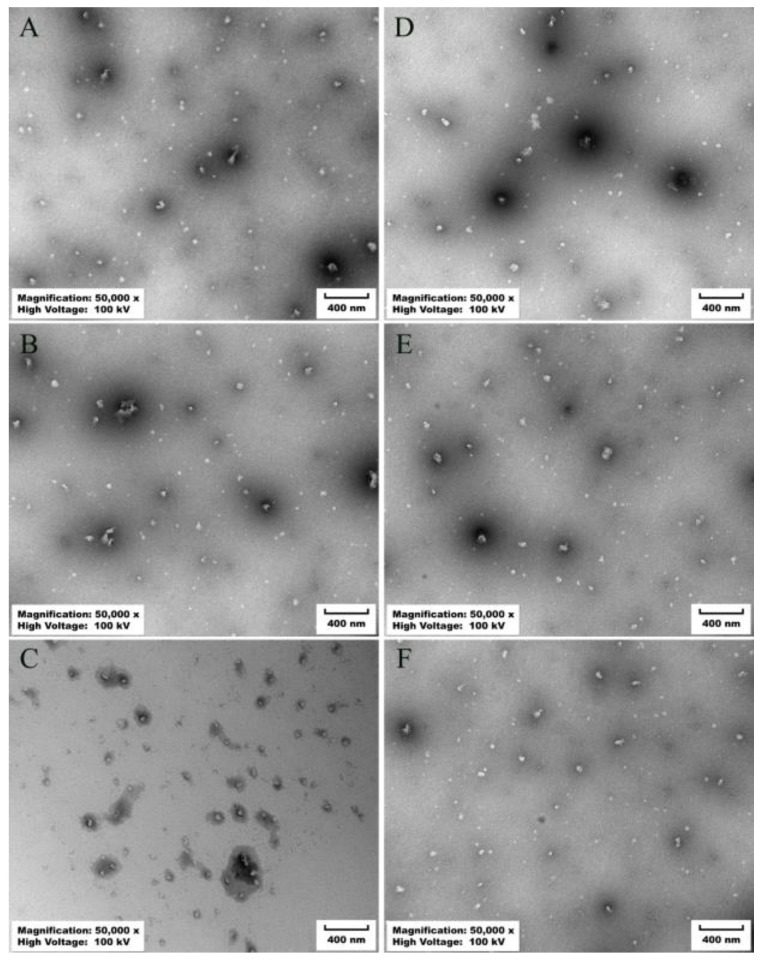
Transmission electron microscope morphology of different samples. (**A**) GWP−SIF nanoparticles; (**B**) GWP−SIF nanoparticles after simulated gastric digestions; (**C**) GWP−SIF nanoparticles after simulated intestinal digestions; (**D**) PGWP−SIF nanoparticles; (**E**) PGWP−SIF nanoparticles after simulated gastric digestions; (**F**) PGWP−SIF nanoparticles after simulated intestinal digestions.

To exert biological activity in the human body, bioactive compounds must undergo preliminary digestion and metabolism. Typically, compounds are digested through simulated stomach and simulated intestine processes before being absorbed through the epithelial cells of the small intestine in the human body [46]. The particle size, zeta potential, and microstructure of the nanoparticles were assessed during the initial, stomach, and small intestine phases. When subjected to simulated gastric fluid, an increase in particle size was observed, with GWP-SIF nanoparticles increasing from 207 nm to 457 nm and PGWP-SIF nanoparticles from 136 nm to 325 nm (Figure 6A). This increase was attributed to the presence of protein-rich particle aggregates. Transmission electron microscopy analysis revealed that both nanoparticles in the initial dispersion exhibited a relatively small size and a uniform distribution within the system (Figure 7). Importantly, particle aggregation was evident after in vitro simulated gastric digestions, supported by particle size measurements (Figure 6A) and microscopy results (Figure 7). These observations may be attributed to variations in the aqueous phase composition of the simulated gastric fluids, including changes in pH, ionic strength, and enzymatic activity [47]. The increase in nanoparticle size from 136 nm to 325 nm, accompanied by blurred boundaries and slight aggregations observed during simulated gastric digestion, indicates that PGWP-SIF nanoparticles have the potential to inhibit digestion in the gastric stage. Furthermore, the absolute value of zeta potential decreased during the gastric phase (Figure 6B). This reduction in zeta potential in the stomach phase can be attributed to the highly acidic conditions and increased ionic strength of the simulated gastric fluids, resulting in a diminished electrical charge and facilitating electrostatic screening and ion binding. This enhanced gastric stability is crucial for protecting the encapsulated SIF from degradation in the acidic environment of the stomach, thereby preserving their bioactivity and ensuring their effective delivery to the small intestine.

Unlike the initial phase, a noticeable increase in particle size was observed for both GWP-SIF and PGWP-SIF nanoparticles after digestion in simulated intestinal fluid, resulting in particle sizes of 413 nm and 304 nm, respectively. Microscopic analysis also revealed the presence of particle aggregates. These aggregates are postulated to consist of partially digested protein particles and particles formed from the products of protein digestion [48]. Furthermore, the nanoparticle systems exhibited a shift toward a highly negative charge upon exposure to the simulated intestinal phase. This phenomenon can be attributed to the neutral pH conditions and the presence of anionic bile salts. Improved gastrointestinal stability leading to enhanced bioavailability of SIF may result in increased health benefits associated with its consumption.

### 3.4. SIF Release during In Vitro Digestions 

**Figure 8:**
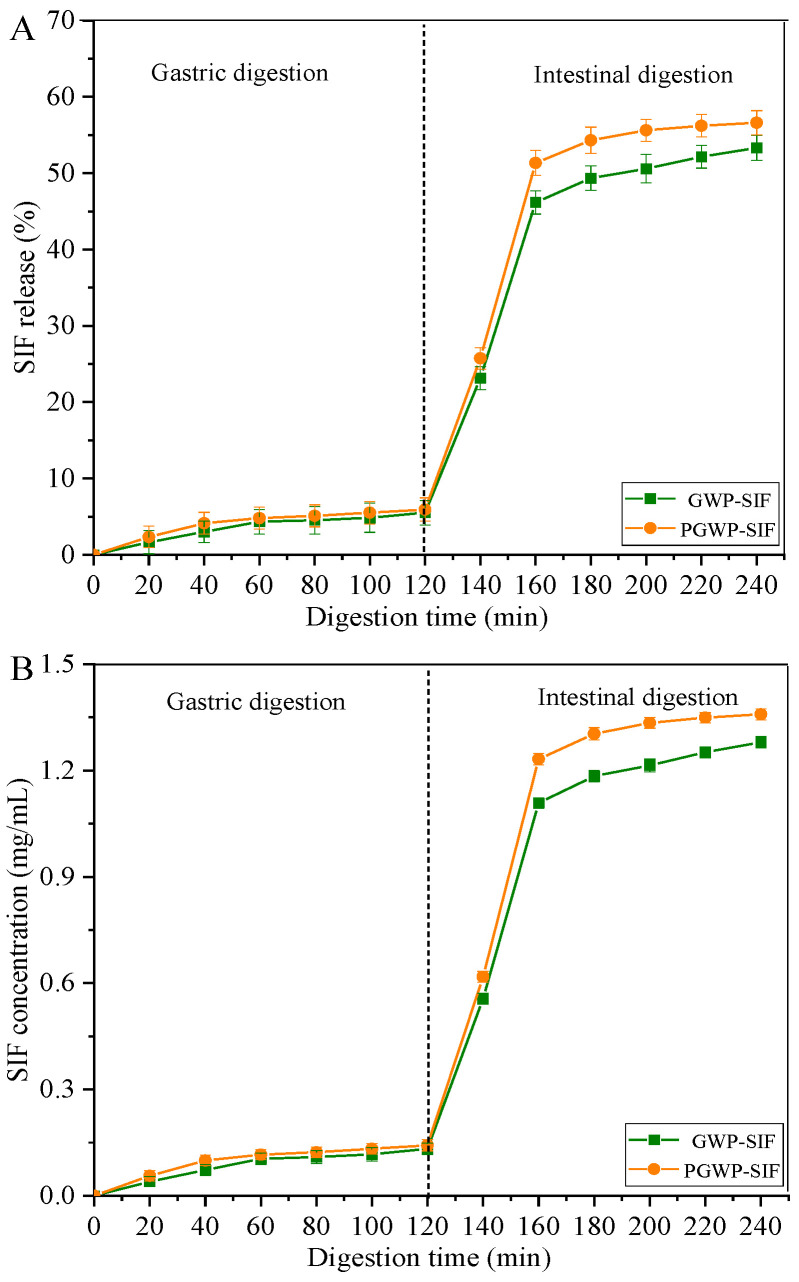
The release (**A**) and concentration (**B**) of SIF from GWP−SIF and PGWP−SIF nanoparticles during in vitro stimulated gastric and intestinal digestion.

The release behavior of SIF from nanoparticle-based delivery systems in the gastrointestinal tract is important for assessing its efficacy. Figure 8A illustrates the release of SIF from both GWP-SIF and PGWP-SIF nanoparticles during in vitro gastrointestinal digestion. During the initial phase of gastric digestion, both nanoparticle systems exhibited slow-release characteristics. After 120 min of gastric phase digestion, the release rate of SIF was 5.52 ± 1.54% for GWP-SIF and 5.93 ± 1.54% for PGWP-SIF nanoparticles, respectively. The low release of SIF after simulated gastric digestion suggests that loading SIF into nanoparticles effectively enhances their gastric digestive stability. The result indicated that both GWP and PGWP as nanocarriers could effectively deliver SIF to the intestine, which was important for SIF digestion and absorption. 

Meanwhile, a substantial increase in SIF release was observed during the intestinal digestion phase, with 53.32 ± 1.66 % and 56.61 ± 1.58 % of SIF released from GWP-SIF and PGWP-SIF nanoparticles, respectively. In simulated gastric digestion, native β-Lactoglobulin and α-Lactalbumin, the predominant components of goat milk whey proteins, largely retained the intact structure. However, hydrolysis progressed rapidly during simulated intestinal digestion by trypsin [23]. In previous studies, various nanoparticle-based delivery systems have been investigated for their release kinetics during simulated gastrointestinal digestion. For instance, Zhong et al. [42] demonstrated a slower release rate of curcumin from whey protein/hyaluronic acid nanoparticles in simulated gastric fluid, attributed to the formation of large electrostatic aggregates at specific pH levels. This led to partial release of curcumin during pepsin digestion on the outer layer of nanoparticles. Additionally, Li et al. [40] reported rapid release of bioactive compounds during small intestinal-juice digestion, attributed to factors such as increased ionic strength inducing charge-shielding effects and trypsin penetration into nanoparticles, facilitating protein hydrolysis and compound release. In our study, the SIF concentrations measured in the final digestion supernatant were 1.280 and 1.359 mg/mL for the GWP-SIF and PGWP-SIF nanoparticles, respectively (Figure 8B). The findings suggested that nanoparticles exhibited sustained and targeted release properties during gastrointestinal digestion, enhancing the bioavailability of SIF [49]. The ability of PGWP-SIF nanoparticles to resist gastric digestion and facilitate the transport of SIF into the small intestine lays the foundation for enhancing the bioavailability of these bioactive compounds.

### 3.5. Cytotoxicity

**Figure 9:**
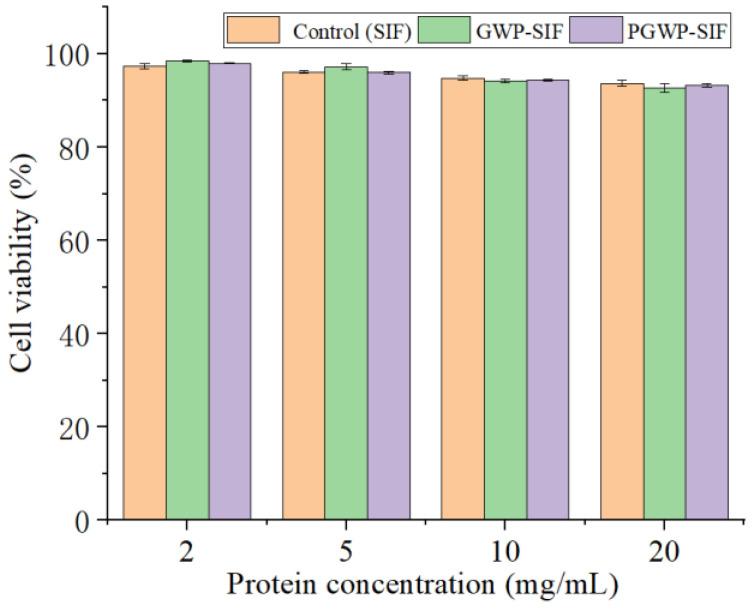
Cytotoxicity results of protein at different concentration on Caco-2 cells by MTT assay.

Assessing biocompatibility in vitro is a crucial step in evaluating the safety of nanoparticles for in vivo applications [50]. This is particularly important for oral delivery systems. Cell viability was assessed using the MTT assay to determine the cytotoxic impact of nanoparticles on Caco-2 cells across varying protein concentrations. A cell viability of >80% indicated that the nanoparticles were non-toxic to the cells [33]. As shown in Figure 9, cell viability consistently exceeded 90% after incubation with SIF nanoparticles at protein concentrations ranging from 2 to 20 mg/mL. The values confirmed that both GWP-SIF and PGWP-SIF nanoparticles exhibited non-toxic characteristics and maintained stability and cytocompatibility within the examined concentration range. The non-toxic characteristics observed for both GWP-SIF and PGWP-SIF nanoparticles have significant implications for their potential applications, particularly in oral delivery systems, and support their biocompatibility and safety profiles. Li et al. [51] had demonstrated that cell viability levels of emulsion gel at three different concentrations were above 97%, indicating excellent biocompatibility and potential for practical applications, a finding that aligns with our observations.

### 3.6. Evaluation of Caco-2 Cell Monolayer

**Figure 10:**
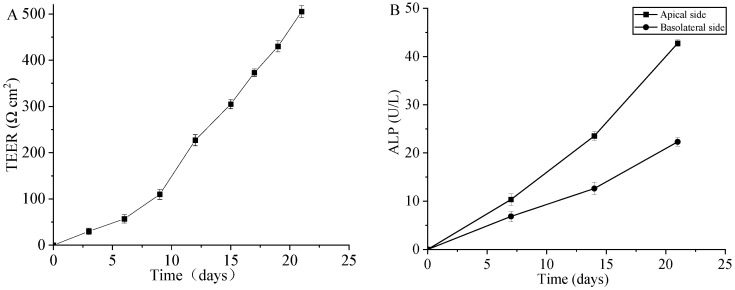
Changes in TEER values of Caco-2 monolayers (**A**) and the activity of alkaline phosphatase (ALP) on both the apical and basolateral sides (**B**) during 21 days of culture.

A Caco-2 cell monolayer is commonly used as an in vitro model to evaluate intestinal absorption of biomaterials [52]. In this study, the trans-epithelial electrical resistance (TEER) values and Alkaline Phosphatase (ALP) levels of the Caco-2 monolayers were used to assess the integrity of the monolayer. As illustrated in Figure 10A, the TEER values exhibited a gradual increase over time in Caco-2 cells. During the initial three days, TEER exhibited a slow rise, indicating cellular proliferation without fusion. However, starting from the seventh day, there was a rapid increase in TEER, signifying the initiation of differentiation. Initially, low TEER values signify the establishment of cell connections, while increasing TEER values over time reflect the maturation and integrity of the cell monolayer. By the fifteenth day, TEER levels reached approximately 300 Ω·cm^2^. Previous research [35] had suggested that TEER values exceeding 400 Ω·cm^2^ indicated successful cell differentiation, rendering them suitable for investigating nutrient transport. With daily monitoring, a significant surge in TEER was observed from the fifteenth day onwards, surpassing 500 Ω·cm^2^ by the twenty-first day. At this point, the membrane had undergone substantial differentiation, making it conducive for transport studies. The observed changes in TEER values indicate the development of tight junctions and barrier function within the cell monolayer, which is essential for accurately assessing bio-material absorption and transport.

ALP activity serves as a marker enzyme of the brush border of intestinal epithelial cells, reflecting the degree of cell differentiation and functional status of the epithelium. The ALP values were presented in Figure 10B. Following a 14-day incubation period, ALP activity was measured at 23.54 ± 1.0 U/L on the apical side and 12.64 ± 1.3 U/L on the basolateral side. Subsequently, after 21 days of incubation, ALP activity was determined to be 42.76 ± 0.7 U/L on the apical side and 22.31 ± 0.9 U/L on the basolateral side. These results suggest a pronounced predominance of ALP production on the apical surface of the cells, signifying the completion of Caco-2 cell polarization and confirming the integrity of the Caco-2 cell monolayer [53]. Therefore, the observed changes in ALP activity levels further confirm the development and maturation of the Caco-2 cell monolayer, indicating its suitability for transport studies.

### 3.7. Transport in Caco-2 Cell Monolayers

**Figure 11:**
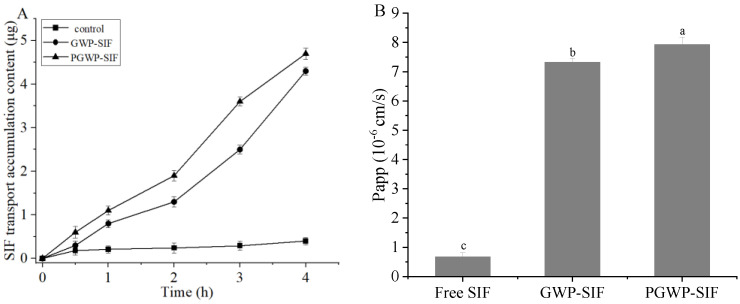
Accumulation transport (**A**) and apparent permeability coefficient (**B**) by Caco−2 cell monolayer with free SIF (control), GWP−stabilized SIF nanoparticle, and PGWP−stabilized SIF nanoparticle. Values with different letters are significantly different (*p* < 0.05).

The transport of free SIF and SIF nanoparticles was investigated, and the results suggested that SIF transport increased with increasing incubation time (Figure 11A). The *Papp* value of free SIF was (6.70 ± 0.16) × 10^−7^ cm/s, which was significantly smaller (*p* < 0.05) than that of GWP-SIF nanoparticles (7.32 ± 0.13) × 10^−6^ cm/s and PGWP-SIF nanoparticles (7.92 ± 0.26) × 10^−6^ cm/s (Figure 11B). Compared with free SIF, the *Papp* values were significantly increased by 10.9- and 11.8-fold for GWP-SIF and PGWP-SIF nanoparticles, respectively. These results indicated that the bioavailability of SIF can be significantly improved by goat milk whey protein-based nanoparticles. Fathima et al. [35] found that delivery systems could improve cellular uptake and transport by increasing the bioaccessibility of active substances. The *Papp* values were significantly higher (*p* < 0.05) for PGWP-SIF nanoparticles compared to those of GWP-SIF nanoparticles, which may be attributed to the smaller particle size of the PGWP-SIF nanoparticles [54]. The nanoparticles may be transported into the cells by endocytosis and subsequently excreted to the basolateral side of Caco-2 cell monolayers by exocytosis [55,56]. Overall, the results showed that SIF nanoparticles were more easily internalized in the intestinal cells than free SIF. 

## 4. Conclusions

A new technique using polymerized goat milk whey protein (PGWP)-encapsulated soy isoflavones (SIF) nanoparticles was developed. The nanoparticles exhibited stability against aggregation over a pH range of 2.0–8.0, salt concentrations of 0–200 mM, temperature fluctuations (4 °C, 25 °C, and 37 °C), and extended storage periods (4 °C, 25 °C, and 37 °C for 30 days), indicating their suitability for use in acidic to neutral food products. During gastric digestion, the release of soy isoflavones (SIF) from PGWP-SIF nanoparticles was 5.93%. In contrast, there was a significant increase in the release of SIF to 56.61% during the intestinal digestion phase. These findings suggest that encapsulating SIF into nanoparticles effectively enhances its gastric digestive stability while facilitating its transport into the small intestine for absorption and utilization. Furthermore, PGWP-SIF nanoparticles demonstrated low cytotoxicity towards Caco-2 cells, exhibiting no adverse effects and indicating their safety for potential use. Importantly, compared to the free form of SIF, encapsulation within PGWP nanocarriers led to a notable increase in the apparent permeability coefficient of Caco-2 cells by 11.8-fold. This enhanced transport across Caco-2 cell monolayers suggests that PGWP nanoparticles may improve the intestinal absorption of SIF, potentially resulting in enhanced bioavailability and therapeutic efficacy.

The PGWP-SIF nanoparticles demonstrate efficacy in extending the shelf life of food products while preserving essential nutrients. Furthermore, the nanoparticles enhance nutrient absorption in dietary supplements, resulting in improved bioavailability. Moreover, the nanoparticles offer precise drug delivery to targeted sites, holding potential for applications in both functional food and pharmaceutical products. Future research could explore the utilization of bovine milk whey protein and goat milk whey protein for encapsulating SIF and assess their impact on nanoparticle characteristics and performance. Additionally, further studies should evaluate absorption kinetics, metabolism, and nutritional functional characteristics of PGWP-SIF nanoparticles under in vivo conditions to enhance their potential for practical applications.

## Figures and Tables

**Table 1:** Standard curve equation of soy isoflavones.

Analytes	Linear Range (μg/mL)	Calibration Curve	R^2^
daidzin	8–40	Y = 37,836 X + 13,575	0.9976
genistin	8–40	Y = 52,171 X + 10,481	0.9975
genistein	8–40	Y = 66,767 X + 3320.3	0.9963
daidzein	8–40	Y = 38,740 X − 5391.4	0.9986
glycitin	8–40	Y = 53,842 X − 2319.4	0.9973

**Table 2:** Storage effects on the particle size and zeta potential of GWP−SIF and PGWP−SIF nanoparticles. Values are presented in mean ± SD.

	Particle Size (nm)						Zeta Potential (mV)					
Days	4 °C GWP-SIF PGWP-SIF		25 °C GWP-SIF PGWP-SIF		37 °C GWP-SIF PGWP-SIF		4 °C GWP-SIF PGWP-SIF		25 °C GWP-SIF PGWP-SIF		37 °C GWP-SIF PGWP-SIF	
1	207 ± 5.5	133 ± 4.6	206 ± 4.5	134 ± 4.8	206 ± 5.8	134 ± 4.2	−24 ± 1.7	−34 ± 1.8	−24 ± 1.4	−33 ±1.7	−25 ± 1.8	−33 ± 1.9
5	210 ± 4.3	135 ± 5.1	212 ± 5.4	135 ± 4.5	214 ± 34	136 ± 3.4	−24 ± 2.0	−33 ± 1.4	−23 ± 1.5	−31 ± 1.5	−23 ± 1.5	−31 ± 1.8
10	214 ± 4.6	135 ± 6.3	218 ± 5.5	137 ± 4.4	219 ± 4.5	139 ± 4.6	−22 ± 1.4	−32 ± 1.6	−22 ± 1.6	−30 ± 1.4	−21 ± 1.4	−30 ± 1.7
15	215 ± 5.5	136 ± 5.0	223 ± 3.9	139 ± 5.7	228 ± 2.7	141 ± 4.5	−20 ± 1.6	−31 ± 1.4	−20 ± 1.7	−28 ± 2.0	−19 ± 1.4	−28 ± 1.5
20	218 ± 4.4	137 ± 5.3	226 ± 4.8	141 ± 6.4	235 ± 4.9	145 ± 3.5	−18 ± 1.7	−29 ± 1.9	−18 ± 1.6	−27 ± 2.0	−17 ± 2.0	−26 ± 1.5
25	220 ± 6.4	138 ± 6.4	228 ± 3.7	144 ± 3.6	242 ± 4.6	153 ± 5.8	−17 ± 1.5	−27 ± 1.5	−16 ± 1.5	−25 ± 1.6	−15 ± 1.6	−23 ± 1.7
30	223 ± 4.3	140 ± 5.5	230 ± 5.6	148 ± 5.5	250 ± 4.7	159 ± 4.5	−16 ± 1.4	−26 ± 1.4	−14 ± 1.7	−24 ± 1.5	−13 ± 1.7	−22 ± 1.9

## Data Availability

Data are contained within the article.
